# Homodimerization of the Death-Associated Protein Kinase Catalytic Domain: Development of a New Small Molecule Fluorescent Reporter

**DOI:** 10.1371/journal.pone.0014120

**Published:** 2010-11-30

**Authors:** Michael Zimmermann, Cédric Atmanene, Qingyan Xu, Laetitia Fouillen, Alain Van Dorsselaer, Dominique Bonnet, Claire Marsol, Marcel Hibert, Sarah Sanglier-Cianferani, Claire Pigault, Laurie K. McNamara, D. Martin Watterson, Jacques Haiech, Marie-Claude Kilhoffer

**Affiliations:** 1 Laboratoire d'Innovation Thérapeutique, Unité Mixte de Recherche 7200, Centre National de la Recherche Scientifique, Université de Strasbourg, Faculté de Pharmacie, Illkirch, France; 2 Laboratoire de Spectrométrie de Masse BioOrganique, Département Sciences Analytiques, Institut Pluridisciplinaire Hubert Curien, Unité Mixte de Recherche 7178, Centre National de la Recherche Scientifique, Université de Strasbourg, Strasbourg, France; 3 School of Science, Xiamen University, Xiamen, Fujian Province, People's Republic of China; 4 Northwestern University, Chicago, Illinois, United States of America; Illinois Institute of Technology, United States of America

## Abstract

**Background:**

Death-Associated Protein Kinase (DAPK) is a member of the Ca^2+^/calmodulin regulated serine/threonine protein kinases. Its biological function has been associated with induced cell death, and *in vivo* use of selective small molecule inhibitors of DAPK catalytic activity has demonstrated that it is a potential therapeutic target for treatment of brain injuries and neurodegenerative diseases.

**Methodology/Principal Findings:**

In the *in vitro* study presented here, we describe the homodimerization of DAPK catalytic domain and the crucial role played by its basic loop structure that is part of the molecular fingerprint of death protein kinases. Nanoelectrospray ionization mass spectrometry of DAPK catalytic domain and a basic loop mutant DAPK protein performed under a variety of conditions was used to detect the monomer-dimer interchange. A chemical biological approach was used to find a fluorescent probe that allowed us to follow the oligomerization state of the protein in solution.

**Conclusions/Significance:**

The use of this combined biophysical and chemical biology approach facilitated the elucidation of a monomer-dimer equilibrium in which the basic loop plays a key role, as well as an apparent allosteric conformational change reported by the fluorescent probe that is independent of the basic loop structure.

## Introduction

Death-Associated Protein Kinase (DAPK) was first identified in a functional screening assay that aimed at identifying genes involved in interferon-γ induced cell death [1]. In addition to its N-terminal kinase domain, DAPK has a calmodulin (CaM)-binding sequence, ankyrin repeats, P-loop motifs, a cytoskeleton binding region, and a death domain (for review see [1], [2]). High-resolution crystallographic structures of various conformations of the DAPK catalytic domain [3], [4], [5], [6] including the apo-form [4] and complexes containing bound nucleotides [4], [5] or small molecule inhibitor fragments [3] have identified structures that are key to catalytic activity [7], [8] as well as novel structural features [4], [7], [9] potentially involved in protein-protein interactions. The DAPK crystallographic structural studies [4] identified a basic loop that is not required for enzymatic activity [7], but has sequence homology to regions of other death protein kinases [4], [7], [9]. A relevant close DAPK family member in this regard is ZIPK [10], which has approximately 80% sequence identity to DAPK in its catalytic domain and possesses such a basic loop sequence. The basic loop region of ZIPK is considered key to functional heterodimer formation between ZIPK and DAPK catalytic domains [9]. This prior work with heterodimer formation led us to postulate that DAPK might self-associate to form homodimers under the appropriate experimental conditions, and that the basic loop region of DAPK might be involved in such self-association.

We report here that noncovalent nanoelectrospray ionization mass spectrometry (nanoESI-MS) analysis of DAPK under different conditions revealed the apparent formation of homodimers. Their absence in a DAPK mutant (DAPKdel) lacking the core sequence of the catalytic domain's basic loop (ΔSRRGVS, residues S52–S57) indicated the importance of this structural feature of DAPK in such a protein-protein interaction. We confirmed the homodimerization by analytical ultracentrifugation and by dynamic light scattering. Based on these initial findings, we screened a fluorescent small molecules based library in order to identify fluorescent binding partners for the DAPK catalytic domain, which could serve as reporters of the protein dimerization state. Fluorescence anisotropy measurements in competition assays indicate spatial independence of the fluorescent probe binding site from those of the peptide substrate or the nucleotides ATP and ADP. The results provide a firm experimental foundation for the future study of DAPK catalytic domain homodimerization and reveal the potential of using fluorescence anisotropy assays to screen for DAPK binding partners.

## Materials and Methods

### Protein expression and purification

The protein expression plasmids pASK-IBA3 (IBA, Göttingen, Germany) encoding the DAPK catalytic domain (DAPKwt, open reading frame residues 1–285) or the mutant protein DAPKdel (ΔS52–S57) were used to produce the respective proteins in *Escherichia Coli* (DH5α) essentially as previously described [7]. Specifically, protein expression was induced at 22°C for 4 h in tryptone soy broth (Biorad, Hercules, CA), bacteria were harvested by centrifugation (15 min, 5000× g), lysed by sonication using a Bioblock scientific vibracell (3 pulses of 15 s at 40% maximum power) and the suspension clarified by centrifugation (20 min, 12000× g). The clarified lysate was applied directly to a high capacity streptactin superflow resin (IBA Göttingen, Germany). In addition to the manufacturer's recommendations, an extra salt wash with three column volumes of the high salt washing buffer (100 mM TrisHCl pH 8, 500 mM NaCl) was performed. Using a 10 kDa cut-off concentrator (Sartorius, Göttingen, Germany) the elution buffer (100 mM TrisHCl pH 8, 150 mM NaCl, 1 mM EDTA, 2.5 mM desthiobiotin) was exchanged with the storage buffer (20 mM TrisHCl pH 7.5, 250 mM NaCl, 1 mM EDTA, 1 mM DTT), and the final volume adjusted in order to achieve protein concentrations between 200 and 700 µM. The concentrations were determined using a NanoDrop spectrometer (Thermo Scientific, Waltham, MA) and a molar extinction coefficient in water of 31400 M^−1^ cm^−1^ at 280 nm for DAPKwt and DAPKdel. Protein preparations were analyzed by nanoESI-MS mass measurement under denaturing conditions as described below and by capillary electrophoresis (Bioanalyzer, Agilent Technologies Santa Clara, CA). The aggregation state homogeneity of the protein solutions was examined by dynamic light scattering measurements (Dynapro Model 801, Wyatt Technology Europe GmbH, Dernbach, Germany).

### Nanoelectrospray ionization mass spectrometry (nanoESI-MS) for the study of noncovalent complexes

Prior to any mass spectrometry experiment, protein buffer was exchanged against a 10 mM ammonium acetate (NH_4_Ac) solution at pH 8.8 using microcentrifuge gel filtration columns (Zeba 0.5 ml, Thermo Scientific, Rockford, IL). Protein concentration was determined spectrophotometrically. ADP solution was desalted using a 1 ml anionic exchange column (HiTrap Q-Sepharose, GE Healthcare, Little Chalfont, UK) in order to exchange sodium counter-ions against volatile ammonium ions. The ADP concentration was then determined spectrophotometrically and magnesium acetate was subsequently added to this solution to reach a 1∶1 ADP: Mg^2+^ molar ratio.

NanoESI-MS measurements were carried out on an electrospray time-of-flight mass spectrometer (LCT, Waters, Manchester, UK) equipped with an automated chip-based nanoESI source (Triversa Nanomate, Advion Biosciences, Ithaca, NY) operating in the positive ion mode. External calibration was performed with the multiple charged ions produced by a 2 µM horse heart myoglobin solution diluted in a 1∶1 (v/v) water: acetonitrile mixture acidified with 1% (v/v) formic acid.

Purity and homogeneity of DAPKwt and DAPKdel were first assayed in denaturing conditions by diluting the proteins to 2 µM in a 1∶1 (v/v) water/acetonitrile mixture acidified with 1% (v/v) formic acid. Analyses in non-denaturing conditions were then performed by diluting proteins to 5 µM in NH_4_Ac buffer at pH 8.8 (adjusted with ammonia). Different NH_4_Ac concentrations were used to test their influence on the protein's oligomerization state. In order to investigate the influence of ADP-Mg on the oligomerization state of the protein, titration experiments were performed using a fixed concentration of DAPKwt (5 µM) and increasing amounts of ADP-Mg. Experiments were realized after careful optimization of instrumental parameters. Particularly, the pressure in the first pumping stage was raised up to 6 mbar using a throttling valve and the acceleration voltage applied on the sample cone was set to 100 V. Data analysis were performed with MassLynx 4.1 (Waters, Manchester, UK).

### Production of the chemical library

#### General considerations and characterization of fluorescent peptides

The design and the combinatorial synthesis of the fluorescent peptides based library followed the previously described procedures [11]. All chemicals were obtained from commercial suppliers and used without any further purification. Lissamine Rhodamine B sulfonyl chloride was purchased from Acros, Rink Amide resin (100–200 mesh) and Fmoc-amino acids from Novabiochem.

The RP-HPLC analyses were performed on a Chromolith SpeedROD RP18 (4.6×50 mm) column using a water/acetonitrile linear gradient (0–100% B in 5 min, 7 mL/min, 220 and 254 nm). The following buffers were used: (eluent A) water containing 0.1% TFA by volume; (eluent B) acetonitrile containing 0.1% TFA by volume. ESI-TOF (electrospray time of flight) spectra were recorded on a Perseptive Biosystem Mariner 5155 spectrometer. The m/z range 200–2100 was scanned using an ion-spray voltage of 4500 V. The nozzle was ranged between 30 and 60 V.

Steady-state absorption spectra were recorded on a NanoDrop spectrometer (Thermo Scientific, Waltham, MA). Steady-state fluorescence spectra were obtained on a Fluorog spectrofluorometer (Jobin Yvon, USA), with 2 nm excitation and emission bandwidths (Supporting Information Figure S1). The excitation wavelength was set at 540 nm. The concentrations were adjusted to an absorbance less than 0.1, in order to keep proportionality between the absorption and the fluorescence intensities of the solutions. All spectra were corrected for lamp intensity variations and background.

#### Synthesis of Lissamine Rhodamine B peptides ortho/para-CHPO 187-3-H11

Both peptides were synthesized on an acid-labile Rink resin [12] (0.7 mmol/g) using standard Fmoc/*tert*-Butyl protocols [13]. The following L-amino-acids were used for the synthesis: Fmoc-Orn(Boc)-OH, Fmoc-2-naphtylalanine-OH, Fmoc-Arg(Pbf)-OH and Fmoc-b-Ala-OH. Coupling reactions (3 h) were performed with 3 equiv. of the appropriate amino-acid using a HOBt/DIC (3/3 equiv.) activation in DMF. Fmoc group was removed using a 80/20 (v/v) DMF/piperidine solution (2×10 min). Following each coupling, the resin was washed with DMF (3×), CH_2_Cl_2_ (3×), MeOH (1×) and CH_2_Cl_2_ (3×). After peptide chain elongation and α-NH_2_- deprotection, the Lissamine Rhodamine B sulfonyl chloride (2 equiv.) was reacted with the resin in presence of DIEA (2 equiv.) in dichloromethane for 5 h. Final cleavage and deprotection were performed by treating the resin with a freshly made TFA/TIS/H_2_O (95/2.5/2.5, v/v/v) solution for 3h. Crude peptide thus obtained was isolated by precipitation with Et_2_O, dissolved in a 1∶1 acetonitrile/water (1/3) mixture and freeze-dried to red foams. Both *ortho*- and *para*-Lissamine Rhodamine B peptides were isolated by RP-HPLC on a C18 Symmetry Shield column from Waters (19×300 mm, 7 µm) at a flow rate of 10 mL/min.

*Ortho*-CHPO 187-3-H11 (4 mg), t_R_ = 1.85 min, RP-HPLC purity >97% (224 nm), LC-MS (ESI) calcd for C_54_H_69_N_11_O_10_S_2_ 1096.3; found 1096.5. *Para*-CHPO 187-3-H11 (14 mg), t_R_ = 1.98 min, RP-HPLC purity >97% (224 nm), LC-MS (ESI) calcd for C_54_H_69_N_11_O_10_S_2_ 1096.3; found 1096.5.

### High throughput screening (HTS) of a fluorescent peptides based library

Fluorescence polarization assays were performed using a Victor 3 apparatus (Perkin-Elmer Waltham, MA). The Lissamine Rhodamine B peptides based library (UMR 7200 CNRS, Strasbourg, France, Patent WO/2006/003330 [14]) was diluted in the assay buffer (50 mM HEPES, 150 mM KCl, 1 mM MgCl_2_, pH 7.5) to a working concentration of 0.2 µM. 15 µl of these dilutions were transferred to each well of the assay plate (Corning Costar 96-well black polystyrene plates, Model 3686, Corning, Acton, MA). All pipetting in HTS was performed on a Biomek® 2000 (Beckman Coulter, Fullerton, CA). Fluorescent polarization degrees (mFP) were measured at an excitation and emission wavelength of 530 and 610 nm, respectively. 15 µl of the assay buffer with DAPK, at a concentration of 4 µM, were added to each well. For each plate, background correction was performed with blank control wells containing no compounds.

### ATP, ADP and substrate peptide titrations

All titration measurements were performed on a FlexStation 3 (Molecular Devices Union City, CA). The other experimental conditions were described in section **“HTS of fluorescent peptides based library”**. A twofold dilution cascade of the protein between 200 µM and 0.8 µM was prepared and 10 µl of these solutions were dispatched in 96-well assay plates. 10 µl of the probe solution (0.2 µM of CHPO 187-3-H11-*para* in assay buffer) were added to the wells and fluorescent polarization was recorded as specified before. Subsequently, 1 µl of concentrated ATP, ADP or substrate peptide [7] (Tocris Bristol, UK) in assay buffer with 0.1 µM of the fluorescent probe was added to the wells, in order to subsequently obtain the following final concentrations of ATP, ADP or substrate peptide: 0.4, 0.8, 1.6, 3.2, 6.3, 12.5, 25, 50, and 1000 µM (the highest concentration was not performed for the peptide).

### Titrations under monomeric and dimeric conditions

Titrations followed the same protocol as described under **“ATP, ADP and substrate peptide titrations”,** but under buffer conditions analogous to the ones listed under “nano-ESI-MS for the study of non-covalent complexes”. For investigations of the monomeric and the dimeric forms, NH_4_Ac concentrations were kept constant at 250 mM and 5 mM, respectively. To induce gradual monomerization of DAPK catalytic core, the NH_4_Ac concentration was stepwise augmented (5, 10, 25, 50, 100 and 250 mM) by the addition of a concentrated NH_4_Ac stock solution (1 M).

To determine the Kd values of γ-[6-Aminohexyl]-ATP-Atto495 (Jena Bioscience, Jena, Germany) its concentration was set to 0.1 µM, while the rest of the conditions were kept identical to the ones described above. Fluorescence polarization was recorded at an excitation and emission wavelength of 493 nm and 520 nm respectively.

### Analytical ultracentrifugation (AUC) and dynamic light scattering (DLS) measurements

Sedimentation velocity experiments were performed in a Beckman-Coulter XL-I analytical ultracentrifuge (Beckman-Coulter, Brea, CA) at 4°C and 50.000 rpm. Absorbance scans were taken at 280 nm every 3 min. AUC was performed for DAPKwt in 5 mM, 50 mM and 250 mM NH_4_Ac, pH 8.8 at protein concentrations of 0.22 µM (for 50 mM and 250 mM ammonium acetate) and 0.3 µM for 5 mM NH_4_Ac). The program Sednterp was used to calculate the partial specific volume [15] using the amino acid composition. The sedimentation data were analyzed with program Sedfit [16] using the continuous c(s) distributions. Buffer density (d) and viscosity (η) under the different conditions were as follows: ammonium acetate 5 mM (d = 1.0002 g/cm^3^, η = 0.0157 Poise); 50 mM (d = 1.002 g/cm^3^, η = 0.0158 Poise); 250 mM (d = 1.010 g/cm^3^, η = 0.016 Poise).

DLS data was obtained on a Dynapro Model 801 (Wyatt Technology Europe GmbH, Dernbach, Germany). Samples were prepared as for AUC analysis. Ten measurements were performed on each sample. Data were analyzed using the instrument software. The time-dependent intensity fluctuations of the scattered light led to an estimation of the average protein particle size.

### Gyration and Stockes radii

The program HYDROPRO (version 7c [17]) was used to calculate the gyration (R_g_) and Stockes radii of DAPK catalytic subunit monomers and dimers using the atomic coordinates present in the PDB files 1JKK and 1JKT, respectively [4]. An AER value of 3.2 Angströms was used to construct the primary hydrodynamic particle. Otherwise default program values were used.

## Results

### Homodimerization of DAPK catalytic domain

**Figure 1:**
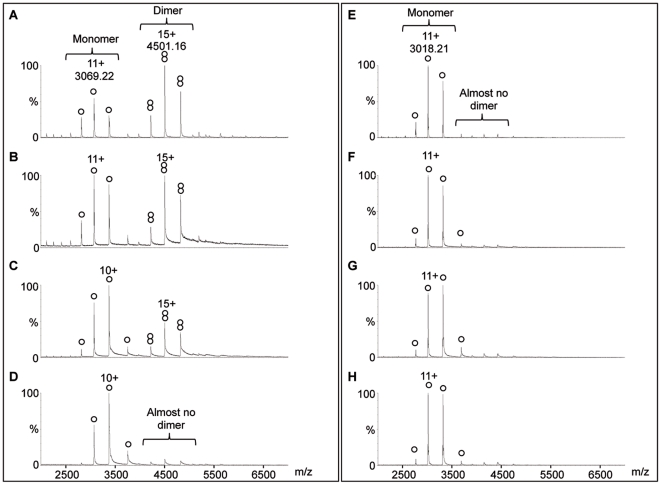
Noncovalent mass spectrometry analysis of (A–D) DAPKwt and (E–H) DAPKdel. Proteins were diluted to 5 µM in NH_4_Ac buffer at (A, E) 5 mM, (B, F) 50 mM, (C, G) 120 mM and (D, H) 250 mM. (○) and (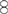) are related to monomeric and dimeric DAPK, respectively.

The question of DAPK catalytic domain (named DAPK in this report) oligomerization state was addressed using automated chip-based nanoESI-MS. DAPKwt (open reading frame residues 1–285) was first analyzed under denaturing conditions, revealing the presence of a species with a molecular mass (33751.1±0.1 Da) in agreement with that calculated from the open reading frame (ORF) amino acid sequence minus the N-terminal methionine (33750.4 Da). A second, less intense ion distribution is also observed with a molecular mass of 33830.7±0.3 Da. DAPKwt was also analyzed under non-denaturing conditions, revealing two ion distributions in the mass ranges m/z 3000–4000 and m/z 4000–5500, respectively (Fig. 1A). The former corresponds to the 10+ to 12+ charge states of monomeric DAPKwt (measured MW = 33750±1 Da), while the latter is assigned to the 14+ to 16+ charge states of dimeric DAPKwt (measured MW = 67501±1 Da). These results indicate a monomer to dimer equilibrium of DAPKwt.

By the same MS based method and by analytical ultracentrifugation, the equilibrium was shown to be sensitive to the buffer ionic strength. In fact, the oligomerization state of DAPK is shifted towards the monomeric form of the protein when the buffer concentration is increased from low to high concentrations of ammonium acetate (from 5 mM to 250 mM NH_4_Ac) (Fig. 1A to 1D and Table 1). In a physiological-like buffer (assay buffer of fluorescent measurements, 50 mM HEPES, 150 mM KCl, 1 mM MgCl_2_, pH 7.5), the protein population is mostly in a dimeric form as shown by dynamic light scattering (DLS) (Table 2). The indicated hydrodynamic radius provides an estimate of the average particle size and the percentage of polydispersity is an index of particle size uniformity. DAPK experimental hydrodynamic radius value in buffer favoring the monomeric form of the protein (R_He_ = 2.13 nm) is within the calculated gyration (R_g_) and Stockes (R_Hc_) radii values calculated using the atomic coordinates of the monomeric form (R_g_ = 2.04 nm and R_Hc_ = 2.65 nm, Table 2). It is closer to the gyration radius than to the Stockes radius. On the other hand, the hydrodynamic radius determined from experiments performed under conditions favoring the protein dimeric form (R_He_ = 3.63–3.68 nm) is very close to the calculated Stockes radius (R_Hc_ = 3.58 nm). It is larger than the gyration radius calculated for the dimer (R_Hc_ = 2.89 nm). Observed differences between experimental and calculated values are generally attributed to hydration effects. In the case of DLS measurements, some uncertainty is also related to the difficulty in polydisperse sample analysis. Nevertheless, values obtained from DLS measurements are in the interval of the calculated gyration and Stockes radii and data are in agreement with the existence of monomeric and dimeric forms of DAPK catalytic subunit.

**Table 1:** Analytical centrifugation of DAPKwt in ammonium acetate buffer (NH_4_Ac, pH 8.8) at different concentrations (pH 8.8).

	s_20,w_ [S]	SD s_20,w_ [S]	calc. M [kDa]	Fraction of species[%]
**NH_4_Ac 5 mM**	2.72	0.038	36.0	17.3
	3.76	0.056	65.9	75.8
**NH_4_Ac 250 mM**	2.76	0.082	33.1	54.8
	4.11	0.112	53.4	34.8

s_20,w_  =  sedimentation coefficient of major species observed corrected to 20°C; SD s_20,w_  =  standard deviation of s_20,w_; Calc. M  =  calculated molecular mass of species.

**Table 2:** Dynamic light scattering (DLS) parameters for DAPKwt in ammonium acetate (NH_4_Ac, pH 8.8) or HEPES Buffer (50 mM HEPES, 150 mM KCl, 1 mM MgCl_2_, pH 7.5).

	R_He_ [nm]	% Polydispersity
**HEPES Buffer**	3.63	16.4
**NH_4_Ac 5 mM**	3.68	39.7
**NH_4_Ac 250 mM**	2.13	22.9

R_He_ represents the hydrodynamic radius obtained from DLS data analysis. Hydrodynamic radii in the table can be compared to gyration (Rg) and Stockes (R_Hc_) radii calculated from the atomic coordinates using the HYDROPRO program as indicated under Materials and Methods. Calculated gyration radii (R_g_) of the monomeric and dimeric forms of DAPKwt are 2.04 nm and 2.89 nm, respectively. Calculated Stockes radii (R_Hc_) for the monomer and dimer are 2.65 nm and 3.58 nm, respectively.

The observed shift from dimeric to monomeric DAPKwt with increasing NH_4_Ac concentrations suggests that the protein-protein association involves electrostatic interactions, which are sensitive to the ionic strength of the medium. Moreover, as the ionic strength is similar in the HEPES buffer and the 250 mM NH_4_Ac buffer, the fact that the protein is mostly in a dimeric form in HEPES buffer and mostly in a monomeric form in NH_4_Ac buffer, suggests a change of its protonation state between pH 7.5 (HEPES buffer) and 8.8 (NH_4_Ac buffer).

### Involvement of DAPK basic loop in the homodimerization process

The ionic strength dependent monomer to dimer equilibrium of DAPK wt prompted us to investigate the role of a basic loop most likely involved in the dimer interface. The loop is located in the DAPK-family fingerprint region (see Figure S2). A mutated catalytic domain (DAPKdel) missing a portion of the basic loop sequence was made by excising the amino acid sequence SRRGVS between S52 and S57 (see Figure S2). DAPKdel was analyzed by noncovalent nanoESI-MS under experimental and instrumental conditions identical to those used for DAPKwt. Analysis under denaturing conditions revealed single polypeptide chain weights of 33108.8±0.7 and 33188.7±0.4. Interestingly, under non-denaturing conditions, DAPKdel is only detected as a monomer (MW = 33108±1 Da; Fig. 1E to 1H), even under low ionic strength conditions previously shown to promote DAPKwt dimerization.

### High Throughput Screening (HTS) of a fluorescent peptides based library

Homodimerization processes are difficult to follow. Fluorescence techniques may be applied if an internal or external fluorescent group, adequately positioned to serve as reporter group, is present. Sometimes, a single tryptophan residue, that can be native or introduced by genetic engineering, may be sufficient to sense such conformational changes [18]. However, as the presence of a unique, ideally positioned tryptophan residue is not a common feature of most proteins, we devised a more general strategy to find a fluorescent probe that binds to the protein with properties relying on the conformation of the protein.

This strategy involves three steps:

1. The design and the synthesis of a fluorescent compounds library using known chemical scaffolds that exhibit low specificity for any given protein;
2. Screening the fluorescent compounds library by fluorescence polarization measurements in order to successfully fish out at least one probe. This technique allows one to set up a “mix and read” assay that readily pinpoints the probe that interacts with the protein;
3. Characterization of the probe binding properties in order to establish that they fulfill our requirements and exhibit a change upon protein oligomerization.

**Figure 2:**
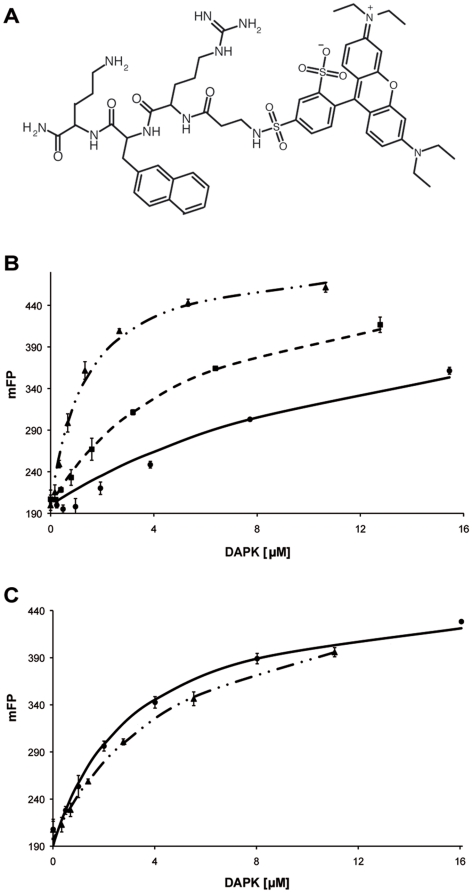
Ability of the fluorescent probe to monitor dimerization of DAPK catalytic core. A) Chemical structure of CHPO 187-3 H11-para, which was selected as best hit in the primary and secondary screening assays (absorption and fluorescent spectra of the probe are provided in Supporting Information, Figure S1). B) Binding of CHPO 187-3 H11-para (0.1 µM) to DAPKwt at different concentrations of NH_4_Ac (• 5 mM, ▪ 100 mM and ▴ 250 mM). Fluorescence polarization (mFP) is plotted *versus* protein concentration. mFP corresponds to the polarization degree of the fluorescent probe ×10^3^. Error bars indicate the standard deviation of four independent titrations. C) Same than in B) but with DAPKdel.

In order to identify molecular probes allowing DAPK oligomerization studies, we screened a chemical library of 1388 compounds tagged with either the *ortho*- or the *para*-isomers of the fluorophore Lissamine Rhodamine B [11] by fluorescence polarization measurements. The measured Z' score indicating the robustness of the assay was always higher than 0.5 (similar to values obtained for other screening experiments performed on the screening platform (http://www.pcbis.fr/)) [11]. After bulk analysis, the 14 compounds (1% hit rate) showing the highest binding affinity were selected for the validation phase. Thereby, purified DAPK catalytic domain was titrated with the identified hit molecules and the resulting fluorescence polarization was recorded at each concentration. These results subsequently allowed us to fit binding curves and to calculate Kd values for each of the molecules assayed. Based on the determined affinity for DAPK and on the compound's convenience to be re-synthesized, the molecule CHPO 187-3-H11-*para* (further on called probe) was selected for all subsequent investigations (Fig. 2A). Probe absorption and fluorescence spectra are shown in Figure S1.

### Interaction of monomeric and dimeric DAPK with the selected probe

In order to assess the fluorescent probe binding affinity to DAPK, the secondary screening assay described above was repeated under buffer conditions favoring either dimeric or monomeric DAPKwt. Titration experiments were performed for both DAPKwt and DAPKdel in 5, 100 and 250 mM NH_4_Ac, pH 8.8. Monitoring fluorescence polarization evidences that the binding affinity of the probe for DAPKwt increases with higher NH_4_Ac concentrations (Fig. 2B). In fact, Kd values calculated from these binding curves are 16.5±1.2 µM and 1.5±0.2 µM under buffer conditions favoring the dimer (5 mM NH_4_Ac) and the monomer (250 mM NH_4_Ac), respectively. In contrast, in the presence of DAPKdel, which was shown to be monomeric regardless of the NH_4_Ac concentration, the probe does not display such an ionic strength dependent binding affinity change (Fig. 2C). Indeed, the Kd value, close to the Kd value of the DAPKwt in the monomer form varies only from 3.4 µM at 5 mM NH_4_Ac to 5.5 µM at 250 mM NH_4_Ac. Altogether, these results clearly show a modified interaction between the probe and the protein depending on its oligomerization state. The fluorescent probe thus appears to be a convenient tool to explore the dimerization state of DAPK's catalytic core.

In a more physiological buffer (50 mM HEPES, 150 mM KCl, 1 mM MgCl_2_, pH 7.5) corresponding to the ionic strength of roughly 200 mM NH_4_Ac, the Kd of the fluorescent probe for DAPKwt is 30.9±2.8 µM, a value close to the affinity expected for probe binding to the dimeric form of DAPKwt (16.5 µM in 5 mM NH_4_Ac, pH 8.8).

### Localization of the probe binding site and modulation of its binding affinity by ATP and ADP

In order to address the question of where the probe binds to the protein, competitive titrations were carried out to verify whether the probe shares the same binding pocket as the known substrates or products of the kinase, *i.e.* ATP, ADP or the substrate peptide [8].

**Figure 3:**
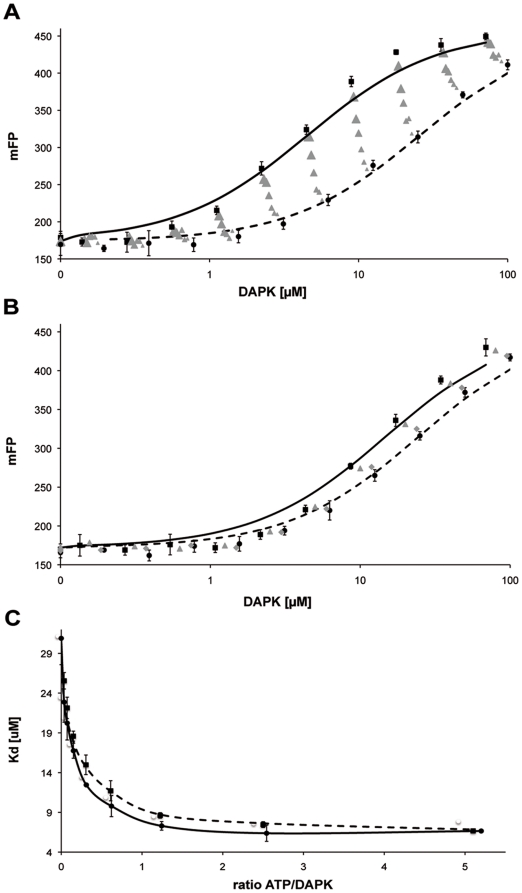
Titration curves showing changes in fluorescence polarization degree as a function of the concentration of DAPKwt in HEPES Buffer pH 7.5 (50 mM HEPES, 150 mM KCl, 1 mM MgCl_2_, pH 7.5). mFP corresponds to the polarization degree of the fluorescent probe ×10^3^. Error bars indicating the standard deviation were calculated from four independent titrations. Error bars are only shown for the most outer curves for sake of figure clarity. A) ATP-titration at the following total concentrations of ATP: • 0 µM; from ▴ to ▴ 0.4, 0.8, 1.6, 3.2, 6.3, 12.5 µM and ▪ 25 µM. The graphs for ATP concentrations of 50 µM and 1000 µM are not shown, as they are congruent with the one at 25 µM. ADP titrations lead to similar results and are not shown. B) Substrate peptide titration. Analogous to the ATP-titration for peptide concentrations of: • 0 µM; 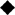 0.8 µM • 6.3 µM; ▪ 50 µM. Only a representative number of titration curves are shown to keep the graphs well distinguishable from each other. C) Fitted Kd values of the fluorescent probe as a function of the stoichiometric ratio of ATP/DAPK (–•–) and ADP/DAPK (- - ▪ - -), respectively.

A sequential multilayered assay in the buffer (see materials and methods), corresponding to a total of 960 different conditions per 96-well plate, was thus developed. For the assay's first layer, 0.1 µM of the probe were incubated with a geometrical concentration range (0 to 100 µM) of DAPK and the fluorescence polarization of Lissamine was recorded. This titration experiment was then repeated in the presence of increasing amounts of ATP, ADP or substrate peptide (0.4, 0.8, 1.6, 3.2, 6.3, 12.5, 25, 50 and 1000 µM). Fig. 3A and 3B show the graphical representation of the procedure's output for ATP and substrate peptide titrations, respectively. The results clearly show that neither ATP nor the substrate peptide is able to chase the probe away from its binding site, as this would lead to a right shift of the titration curves with increasing ATP or substrate peptide concentrations. Identical results were obtained with ADP replacing ATP. Fig. 3A and 3B show that the probe binding affinity of DAPKwt is improved in the presence of ATP (ADP gives results similar to ATP and is not represented) and substrate peptide, respectively. Such an observation suggests a coupling mechanism between the probe binding site and the ATP and substrate peptide binding sites.

In an attempt to represent the described data in a clear overview, we modeled the experimental data using the general framework described by Roehrl et al. [19]. From our data points, we established binding curves corresponding to fluorescence polarization changes as a function of ATP/DAPK or ADP/DAPK concentration ratios. Kd values obtained from these curve fittings were then plotted as a function of the stoichiometric ratio of either ATP or ADP to DAPK (Fig. 3C). The graphic clearly shows the similarity of the effects of ATP and ADP, as corresponding Kd values of both titrations are comparable. This representation also allows visualizing the magnitude of the induced effect. In the absence of either ATP or ADP, the probe Kd value was determined to be 30.9±2.8 µM. ATP or ADP increases the probe affinity for DAPK. A maximal effect was achieved at ATP or ADP to DAPK ratios higher than two, which led to a Kd value of the probe of 6.40±0.54 µM. Both ATP and ADP have the ability to increase the probe affinity roughly five times.

### ATP/ADP induced monomerization of DAPK's catalytic core

Considering on the one hand the probe's ATP- or ADP-dependent increase in affinity for DAPK and on the other hand its stronger binding to monomeric DAPK, one can infer the following: 1. the monomeric form of the catalytic domain has a higher affinity for ATP and 2. ATP or ADP can shift the monomer/dimer equilibrium towards a higher abundance of the monomeric form.

**Figure 4:**
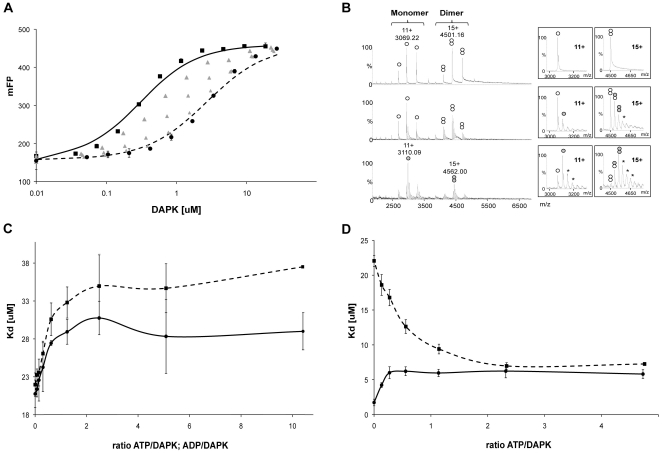
ATP/ADP binding to the catalytic domain of DAPK. A) Binding of the fluorescently tagged ATP (Atto-495-ATP) to the catalytic domain of DAPKwt. mFP corresponds to the polarization degree of the fluorescent probe ×10^3^. One binding curve corresponds to a constant concentration of NH_4_Ac, pH 8.8: • 5 mM; from ▴ to ▴ 25, 50 and 100 mM, ▪ 250 mM. The binding curve of ATP shifts towards the left when the monomeric form is induced by higher NH_4_Ac concentrations. B) Noncovalent mass spectrometry analysis of DAPKwt at 5 µM in 50 mM NH_4_Ac buffer at pH 8.8 either alone (top) or in the presence of 25 µM ADP-Mg (middle) or 100 µM ADP-Mg (bottom). Inserts correspond to enlargements of 11+ and 15+ charge states of monomeric and dimeric DAPKwt, respectively. 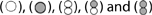 are related to 1∶0, 1∶1, 2∶0, 2∶1 and 2∶2 DAPKwt: ADP-Mg complexes respectively. * represent non-specific ADP-Mg adducts. C) Fitted Kd values of the fluorescent probe as a function of ATP/DAPKdel 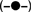 and ADP/DAPKdel 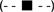 ratios. D) DAPKwt is assayed under conditions favoring monomers (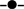 250 mM NH_4_Ac, pH 8.8) or dimers (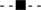 5 mM NH_4_Ac, pH 8.8).

In order to test the inference concerning ATP affinity, Kd-values were determined for a fluorophore labeled ATP (γ-[6-Aminohexyl]-ATP-Atto495) under the buffer conditions promoting the dimeric (5 mM NH_4_Ac, pH 8.8) or the monomeric form (250 mM NH_4_Ac, pH 8.8) of DAPKwt. The results are consistent with the hypothesis. The binding curve is clearly shifted to the left under conditions favoring monomers (Fig. 4A). The Kd value of the labeled ATP for the dimer was estimated to be 3.98±0.22 µM, while the Kd value for the monomer was estimated to be 0.290±0.011 µM.

To further refine our model of ATP/ADP induced monomerization, three further experiments have been performed:

- Noncovalent ESI-MS experiments were carried out with DAPKwt in the presence of increasing amounts of ADP-Mg. Fig. 4B shows that incubation of the protein with five to twenty molar equivalents of ADP-Mg leads to the detection of 1∶1, 2∶1 and 2∶2 DAPK: ADP-Mg complexes (34199±1 Da, 67964±4 Da, 68411±6 Da, respectively). Increasing ADP is accompanied by a shift of DAPK's oligomerization equilibrium towards the monomeric form, consistent with our model of ATP/ADP induced monomerization (Fig. 4B).
- We investigated whether DAPKdel, which exhibits less dimerization, would modify the probe binding upon the addition of ATP or ADP. Multilayered titration matrices were performed with DAPKdel in the physiological-like buffer (50 mM HEPES, 150 mM KCl, 1 mM MgCl_2_, pH 7.5). At ratios of ATP or ADP to DAPKdel higher than two, the Kd values of the probe are maximal for both effectors. The calculated Kd values are 28 µM and 33 µM in the presence of ATP and ADP, respectively (Fig. 4C), starting from a Kd value of 20 µM in absence of ATP or ADP (it should be noted that DAPdel Kd value was 5.5 µM in the 250 mM NH_4_Ac buffer pH 8.8, suggesting an impact of pH on the probe binding to the mutated protein).
- ATP or ADP binding to DAPK appears to trigger two opposite effects that impact the probe affinity. First, there is an apparent increase in affinity of the probe for DAPK due to the dimer to monomer transition. Second, there is an apparent ATP- or ADP-induced decrease in the probe affinity for DAPK in solutions where DAPK is predominately monomeric. We postulate that the latter effect is due to a coupling between the ATP/ADP binding site and the probe binding site in the monomeric form of DAPK catalytic domain. In order to distinguish those two effects, ATP titrations were repeated under experimental conditions favoring either the presence of the monomer or the dimer of DAPKwt (250 mM or 5 mM NH_4_Ac, pH 8.8, respectively). Fig. 4D shows the probe Kd values for either the monomer or the dimer of DAPKwt as a function of the ratio of ATP to the protein. In the monomeric form (250 mM NH_4_Ac, pH 8.8), increasing ATP concentrations induce a decrease of the probe affinity, consistent with an interaction between the ATP binding site and the probe site. When starting from the dimeric state (5 mM NH_4_Ac, pH 8.8), rising ATP concentration induces an increase of the probe affinity, suggesting that ATP binding can also induce a dimer-monomer transition in this same buffer.

## Discussion

Our results show for the first time the homodimerization of DAPK catalytic domain and the apparent key role played by the basic loop structure in this protein-protein interaction. The results are consistent with the proposed model of an equilibrium between monomers and dimers of the DAPK catalytic domain, which can be shifted towards the monomers by the presence of either ATP or ADP. Screening of a fluorescently tagged library of small molecules yielded a new probe that could be used to monitor this equilibrium in solution.

Moreover, the study shows a coupling between the fluorescent probe binding site and nucleotide binding site in solutions where DAPK catalytic domain is mainly in monomeric form.

Removal of the basic loop attenuates the dimer to monomer equilibrium, but allows retention of the apparent coupling between the ATP/ADP binding site and the probe binding site. It should be noted that the equilibrium studied here cannot readily be extended to catalytic activity because the standard conditions used in protein kinase catalytic activity assays are such that, in the case of DAPK, a mixture of monomers and dimers would most likely be present. In contrast, the reagents and conditions defined in this study provide a firm foundation for the future development of screening assays focused on changes brought about by protein-protein interactions.

The approach used in this study demonstrates the utility of combining nanoESI-MS analyses with chemical biology investigations. MS experiments allowed us to detect a monomer/dimer equilibrium of the DAPK catalytic domain. The chemical biology approach permitted the unbiased identification of fluorescently tagged protein ligands that bind to sites not defined *a priori*. The fluorescent traceability of these small molecule reporters, and their non-competitive binding in the case of DAPK, make them well-suited as probes that sense the state of molecular systems under a variety of experimental conditions. By combining those two biophysical approaches, we have identified a molecular tool that is sensitive to the oligomerization state of the DAPK catalytic domain. The probe allowed us to gain insight into the modulation of this monomer/dimer equilibrium. Moreover, the use of a mutated DAPK catalytic domain relaxed the dimerization capability, allowing us to distinguish a modification of the probe binding affinity due to experimental conditions (seen both with the wild type and the mutated core domain of the kinase) or due to dimerization (only seen with the wild type core domain).

## Supporting Information

Figure S1 Steady-state absorption and fluorescence spectra of the fluorescent probe CHPO 187-3-H11 in the various buffers used in this study. (A) Absorption spectra in the assay buffer at pH 7.5 and in NH4Ac 5 or 250 mM at pH 8.8. (blue) (B) Fluorescence spectra in the assay buffer at pH 7.5 (gray), in NH4Ac 250 mM at pH 8.8 (green) and in NH4Ac 5 mM at pH 8.8 (red dashes). Excitation wavelength was at 540 nm. The absorption and fluorescence spectra were normalized to the same maximum amplitude. Experiments were performed at room temperature.
(1.45 MB TIF)

Figure S2 3D-structure of the protein dimer of the catalytic subunit of DAPKwt. The monomers are shown in yellow and blue. The deleted amino acid sequence (SRRGVS) of the mutant protein is highlighted in red in each of the two monomers. The figure was generated with Pymol (DeLano, W.L. MacPyMOL: A PyMOL-based Molecular Graphics Application for Mac OS X (2007) DeLano Scientic LLC, Palo Alto, CA, USA) using the PDB-file 1JKT (Tereshko et al. Nat Struct Biol (2001) vol. 8 (10) pp. 899-907).
(9.46 MB TIF)
