# Supplementary figures and images for: Homodimerization of the Death-Associated Protein Kinase Catalytic Domain: Development of a New Small Molecule Fluorescent Reporter
Source: PLoS One. 2010 Nov 30;5(11):e14120. doi: 10.1371/journal.pone.0014120 (PMC2994711; doi:10.1371/journal.pone.0014120)

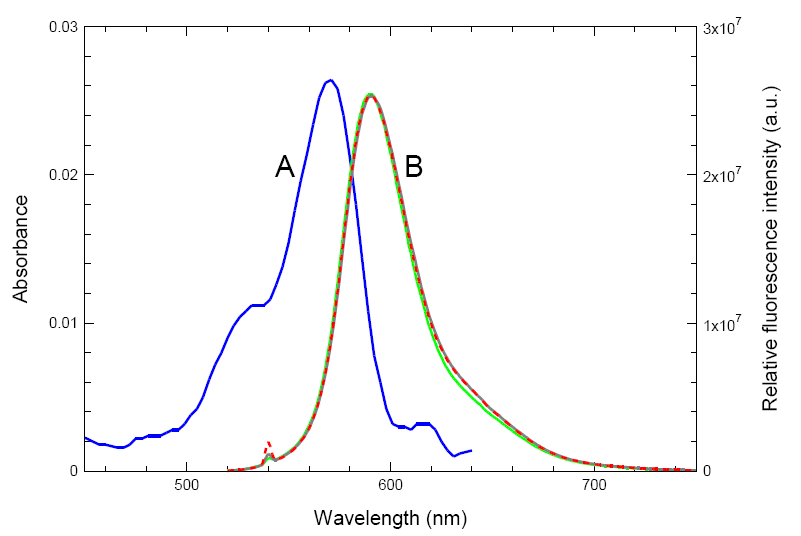

Supplement: Figure S1 — Steady-state absorption and fluorescence spectra of the fluorescent probe CHPO 187-3-H11 in the various buffers used in this study. (A) Absorption spectra in the assay buffer at pH 7.5 and in NH4Ac 5 or 250 mM at pH 8.8. (blue) (B) Fluorescence spectra in the assay buffer at pH 7.5 (gray), in NH4Ac 250 mM at pH 8.8 (green) and in NH4Ac 5 mM at pH 8.8 (red dashes). Excitation wavelength was at 540 nm. The absorption and fluorescence spectra were normalized to the same maximum amplitude. Experiments were performed at room temperature. (1.45 MB TIF) [file pone.0014120.s001.tif]

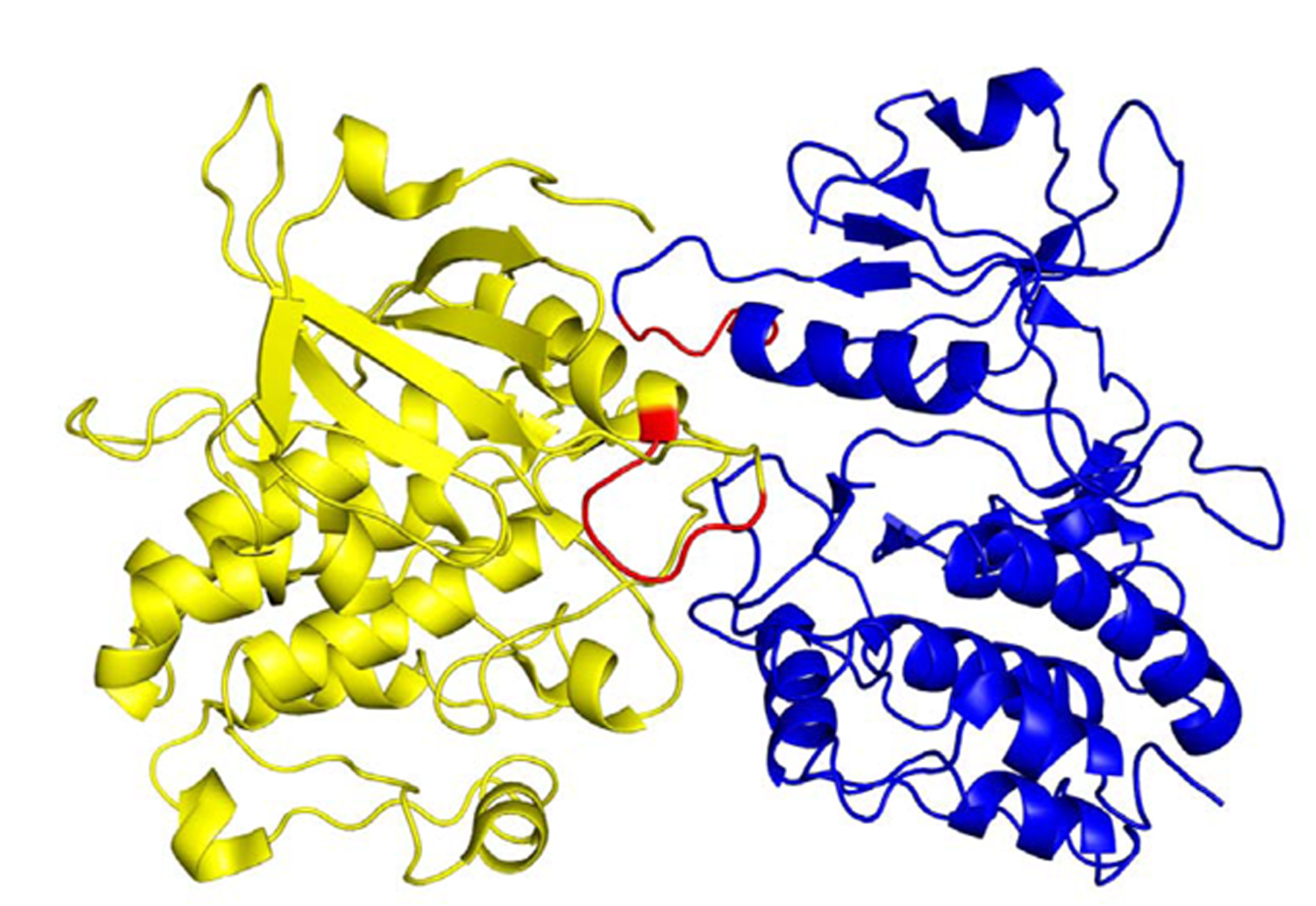

Supplement: Figure S2 — 3D-structure of the protein dimer of the catalytic subunit of DAPKwt. The monomers are shown in yellow and blue. The deleted amino acid sequence (SRRGVS) of the mutant protein is highlighted in red in each of the two monomers. The figure was generated with Pymol (DeLano, W.L. MacPyMOL: A PyMOL-based Molecular Graphics Application for Mac OS X (2007) DeLano Scientic LLC, Palo Alto, CA, USA) using the PDB-file 1JKT (Tereshko et al. Nat Struct Biol (2001) vol. 8 (10) pp. 899-907). (9.46 MB TIF) [file pone.0014120.s002.tif]
